# Assessment of Noninferiority Margins in Cardiovascular Medicine Trials

**DOI:** 10.1016/j.jacadv.2024.101021

**Published:** 2024-06-05

**Authors:** Antonio Greco, Marco Spagnolo, Claudio Laudani, Giovanni Occhipinti, Maria Sara Mauro, Federica Agnello, Denise Cristiana Faro, Marco Legnazzi, Carla Rochira, Lorenzo Scalia, Davide Capodanno

**Affiliations:** Division of Cardiology, Azienda Ospedaliero-Universitaria Policlinico “G. Rodolico-San Marco”, University of Catania, Catania, Italy

**Keywords:** cardiovascular medicine, methodology, noninferiority, noninferiority margin, randomized trials, trial interpretation

## Abstract

**Background:**

Noninferiority trials are increasingly common in cardiovascular medicine, but their reporting and interpretation are challenging, particularly when an absolute risk difference is used as noninferiority margin.

**Objectives:**

This study aimed to investigate the effect of using absolute rather than relative noninferiority margins in cardiovascular trials.

**Methods:**

We reviewed noninferiority trials presented at major cardiovascular conferences from 2015 to 2022 and published within the same period. Based on the actual versus anticipated event rates in the control group, we recalculated the absolute noninferiority margin and re-assessed the trial results. The primary outcome of interest was the proportion of trials with a different interpretation after recalculation. Additionally, we analyzed the conclusion statements of these trials to determine if cautionary notes for the interpretation of study results were included.

**Results:**

We analyzed a total of 768 trials, of which 88 had a noninferiority design and 66 used an absolute noninferiority margin. Of 48 comparisons from 45 trials qualifying for the analysis, 11 (22.9%) had divergent results after recalculation of the absolute noninferiority margin based on the observed rather than anticipated event rate. Ten trials originally claiming noninferiority, did not meet it after the margin recalculation. All of them did not include statements suggesting cautionary interpretation of the study results in the conclusion section. Compared with the other trials, these displayed a larger median difference between anticipated and recalculated noninferiority margins (44.7% [IQR: 38.6%-56.7%] vs 15.3% [IQR: −1.5% to 28.9%]; *P* < 0.001).

**Conclusions:**

Recalculating noninferiority margins based on actual event rates, rather than anticipated ones, led to different outcomes in approximately 1 out of 4 cardiovascular trials, with most divergent trials lacking cautionary interpretation. These findings emphasize the importance of using or supplementing the relative noninferiority margin, particularly in studies with significant deviations between observed and expected event rates. This underscores the critical need for enhanced methodological and reporting standards in noninferiority trials, especially those employing absolute margins.

In evidence-based medicine, randomized controlled trials are critical for advancing science and informing clinical practice.^1^, ^2^, ^3^ However, several types of bias in trial design and conduction may affect their results and interpretation.^4^ Communicating the primary results of a trial at a large international medical conference with simultaneous or subsequent publication in a peer-reviewed medical Journal is another step where substantial bias may occur.^5^, ^6^, ^7^, ^8^, ^9^, ^10^ For instance, the conclusions of a study may be intentionally or unintentionally interpreted and reported with positive connotations despite evidence of neutral or negative results.^11^, ^12^, ^13^, ^14^, ^15^, ^16^, ^17^

These issues can be even more challenging in the setting of a noninferiority design. Experts and regulatory authorities established some critical considerations in this scenario.^18^ The reporting and interpretation of noninferiority trials depend on rejecting or failing to reject a so-called “null hypothesis” (ie, arm A is inferior to arm B) and therefore accepting the corresponding “alternative hypothesis" (ie, arm A is inferior to arm B by less than a certain prespecified treatment effect, also known as noninferiority margin).^11^, ^12^, ^13^, ^14^ In particular, the outcome of a noninferiority trial depends on where the CI of the effect size for a treatment or strategy lies around the noninferiority margin.^18^^,^^19^ Therefore, the choice of the margin represents a key issue for the validity and credibility of a noninferiority trial. To establish an accurate summary estimate of the treatment effect, regulators recommend that previous studies of the active control versus placebo are evaluated and, as appropriate, the effect size is obtained by pooling the available measures with meta-analytic methods, with the final aim to preserve more than a half of the putative effect of the active control versus placebo when selecting the noninferiority margin.^20^^,^^21^

The noninferiority margin is typically prespecified by the investigators either as an absolute risk difference (ARD) or as a relative risk ratio (RRR) of the treatment effect. An absolute margin is preferrable to assess infrequent events or to communicate the risk to an individual patient, while relative metrics are helpful at the population level.^22^^,^^23^ In cardiovascular medicine, absolute metrics are frequently preferred; however, using an ARD margin carries some risk of regression towards noninferiority if the trial terminates with a lower-than-anticipated event rate.^15^, ^16^, ^17^

The reporting and interpretation of noninferiority trials have been the objectives of previous investigation in different medical fields.^24^, ^25^, ^26^, ^27^, ^28^, ^29^, ^30^ In cardiovascular medicine, where noninferiority designs are common for introducing new treatments, a study by Simonato et al. found that about a third of noninferiority trials in interventional cardiology reached different conclusions when using ARD margins compared to RRR margins. However, their focus on coronary stent trials limits broader applicability, and they did not examine how these trials were reported.^30^^,^^31^

Our hypothesis is that noninferiority trials in this field frequently exhibit discordant outcomes based on prespecified vs recalculated ARD margins, and that many of these trials lack adequate cautionary interpretation in their conclusions when the study results are not certain due to lower-than-expected event rates. To fill this gap, we aimed at characterizing the reporting and interpretation of noninferiority trials across the broad field of cardiovascular medicine by addressing the prevalence of different types of noninferiority margin, and by evaluating the impact of using absolute rather than relative noninferiority margins in the context of lower-than-anticipated event rates.

## Methods

### Study selection

In this cross-sectional study, late breaking science sessions from 3 major international annual cardiology conferences (ie, American College of Cardiology, American Heart Association, and European Society of Cardiology) and a large interventional cardiology meeting (ie, Transcatheter Cardiovascular Therapeutics) were scrutinized for studies presented since January 2015 and simultaneously or successively published in extenso in a peer-reviewed medical Journal as of November 10, 2022. This process was aimed at identifying a sample of peer-reviewed and published trials characterized by ample mediatic exposure, likely representing the most cutting-edge research in the field and impacting on practice, with less emphasis on studies with lower potential for dissemination and publication in top-tier journals. The search strategy is described in detail in the appendix (Supplemental Methods 1). The screening results were jointly reviewed by the authors to solve potential discrepancies. All studies had to meet the following inclusion criteria: 1) randomized trials with at least 1 primary analysis powered for noninferiority; and 2) parallel design. Follow-up reports of previously presented or published noninferiority trials, subgroup analyses of noninferiority trials and noninferiority trials with event-driven design were excluded. Studies that did not report information relevant to sample size calculation or determination of the noninferiority margin were also excluded.

### Data collection

All the eligible studies were scrutinized at the full-text level and a set of prespecified variables was extracted and centralized into a dedicated database. For each study, the data collected included details of publication history (ie, journal and timing from presentation), topic (ie, devices or drugs), study design (eg, sample size, analysis, sponsor and involvement in data analysis, endpoint, location, blinding), noninferiority margin scale (ie, ARD or RRR), follow-up interval, outcome (ie, consistency with the pre-specified hypothesis) and results (ie, observed event rate). Noninferiority margins and treatment effect sizes with corresponding CIs were also scrutinized. This study did not require ethical approval as it involved the analysis of publicly available data and did not include any direct interaction with human or animal subjects.

### Assessment of noninferiority margin

The relative frequency of using an ARD or a RRR as noninferiority margin was analyzed. Noninferiority trials using an ARD were further assessed if none of the following cases occurred: 1) the study used an ARD margin, but the results were reported using the upper confidence boundary of a RRR; and 2) the primary endpoint was reported as a continuous variable or event-free rate. To evaluate the impact of using an absolute noninferiority margin we recalculated the ARD margin based on the event rate observed at the end of the trial in the control group (ie, as opposed to the event rate anticipated by the investigators) (Supplemental Figure 1). In brief, for each ARD margin, the corresponding RRR margin was calculated as the ratio between the acceptable rate of events in the control arm (ie, anticipated event rate plus ARD margin of noninferiority) and the anticipated event rate in the control arm.^19^^,^^30^ A new acceptable event rate was then calculated as the product of the corresponding RRR margin of noninferiority and the event rate that was actually observed in the control arm. Finally, a revised ARD margin of noninferiority was calculated as the difference between the new acceptable event rate and the observed event rate in the control arm (Supplemental Figure 1, Supplemental Methods 2). The results were considered “divergent” (primary outcome) from the primary analysis if the upper boundary of the CI for the trial summary estimate (at the level of significance prespecified by the investigators) crossed the recalculated absolute noninferiority margin but did not cross the prespecified absolute noninferiority margin, or vice versa.

### Assessment of trial interpretation

We scrutinized the conclusion sections of trials with diverging results for the presence of cautionary notes, ie, highlighting the risk associated with using an absolute noninferiority margin in the context of a lower-than-anticipated event rate (secondary outcome). Each article was read at the full-text level and analyzed by at least 3 independent investigators and then discussed in a consensus meeting.

### Statistical analysis

Continuous variables are reported as mean ± SD or median (IQR) based on the visual estimation of distribution plots. Comparisons of baseline trial characteristics were made by means of t-Student or Mann-Whitney tests. Categorical variables are reported as frequencies and percentages and comparisons were made by the chi-square or Fisher’s exact tests as appropriate. All the tests were conducted at a 2-sided 5% significance level. All statistical analyses were conducted with R, version 4.0.5 (R Foundation for Statistical Computing). This study followed the STROBE (Strengthening the Reporting of Observational studies in Epidemiology) guidelines for reporting of observational studies, which comprises a checklist of 22 items that were carefully considered during the design, analysis, and reporting of the study (Supplemental Table 1).

## Results

**Figure 1:**
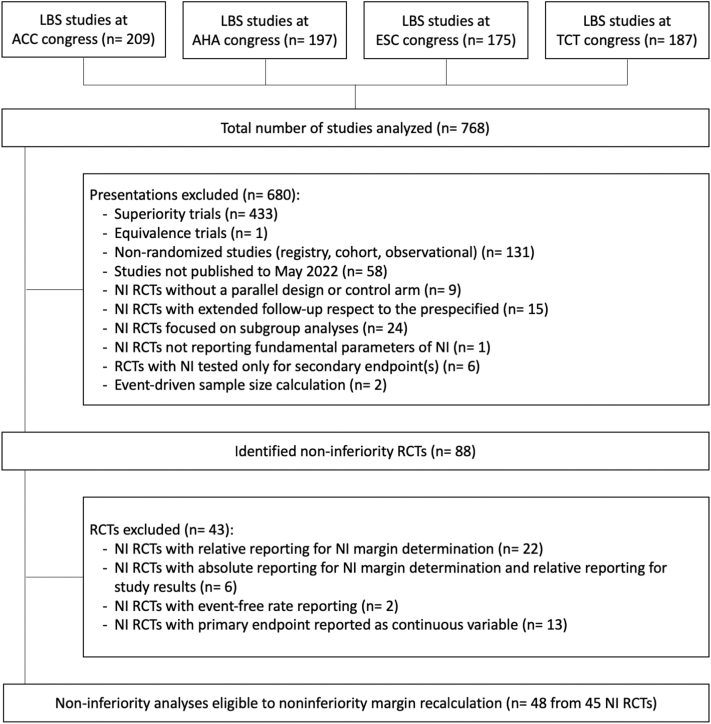
**Study Flow Chart** ACC = American College of Cardiology; AHA = American Heart Association; ESC = European Society of Cardiology; LBS = Late Breaking Science; NI = noninferiority; RCT = randomized controlled trial; TCT = Transcatheter Cardiovascular Therapeutics.

The study flow chart is shown in Figure 1. Of 768 cardiovascular studies presented and published within the study period, 680 were excluded, mainly because they used a superiority design (n = 433, 63.7%) or because they were not randomized (n = 131, 19.3%). A total of 88 (11.4%) randomized trials had a noninferiority design.

### Study characteristics

Key characteristics of the noninferiority trials analyzed are summarized in Supplemental Table 2. All trials had an active comparator and most of them were multicenter (97.6%) and open label (69.4%). The most frequently investigated treatments were devices (59.1%), followed by drugs (30.7%). The median sample size was 1,504 patients (IQR: 761.0-2,471.5 patients) and the median follow-up was 52.1 weeks (IQR: 34.6-56.5 weeks). The primary endpoint was analyzed according to the intention-to-treat principle in 78.4%. Most trials were industry-sponsored (79.5%), and the study sponsor was involved in the trial design or the analysis in 28.4% and 21.6% of cases, respectively.

A positive result consistent with the pre-specified hypothesis of noninferiority was claimed by 81.8% of studies. The median observed event rate in the control arm was generally lower than the anticipated event rate (−17.1%; IQR: −35.8% to 8.7%). The articles corresponding to the trial presentation were published in a total of 14 peer-reviewed Journals covering the fields of cardiovascular and general medicine, often simultaneously (56.8%) with the congress presentation. Trials that were not simultaneously published had a median time from presentation to publication of 235.0 days (IQR: 120.2-333.5 days).

### Noninferiority margin characteristics across trial population

Of the noninferiority trials analyzed, 66 (75.0%) used an ARD as noninferiority margin and 22 (25.0%) used a RRR margin (Table 1, Central Illustration). Compared with trials using a RRR margin, those using an ARD margin more frequently investigated devices (68.2% vs 31.8%; *P* = 0.001) and less frequently were double-blinded (4.5% vs 18.2%; *P* = 0.003). In addition, studies using an ARD margin had a smaller median sample size (1,337.5 [IQR: 527-2,262.8] patients vs 2,134 [IQR: 1,444.5-2,965.0] patients; *P* = 0.005) and a shorter median follow-up duration (52.1 [IQR: 28.0-52.1] weeks vs 69.5 [IQR: 52.0-143.4] weeks; *P* = 0.006). Trials using a RRR had a lower median margin compared to the corresponding median RRR calculated in trials using an ARD (1.4 [IQR: 1.3-1.5] vs 1.5 [IQR: 1.3-1.7]; *P* = 0.036). There were no significant differences in the proportion of studies that claimed noninferiority between the 2 groups (86.4% vs 68.2% for studies using ARD and RRR margins, respectively; *P* = 0.111). There were also no statistically significant differences in the publication timing (*P* = 0.094) and in all the other variables explored (Table 1).

**Table 1:** Study Characteristics According to the Chosen Type of Noninferiority Margin

	RRR Margin (n = 22)	ARD Margin (n = 66)	*P* Value
Study design			
Topic			0.001
Devices	7 (31.8)	45 (68.2)	
Drugs	14 (63.7)	13 (19.7)	
Others^a^	1 (4.5)	8 (12.1)	
Multicenter	21 (95.5)	65 (98.5)	>0.99
Trial design			0.003
Open label	17 (77.3)	45 (68.2)	
Single blind	1 (4.5)	18 (27.3)	
Double blind	4 (18.2)	3 (4.5)	
Sample size	2,134 (1,444.5-2,965.0)	1,337.5 (527-2,262.8)	0.005
Relative margin^b^	1.4 (1.3-1.5)	1.5 (1.3-1.7)	0.036
Follow-up (weeks)	69.5 (52.0-143.4)	52.1 (28.0-52.1)	0.006
Type of analysis			0.79
As treated	0 (0.0)	2 (3.0)	
Intention-to-treat	18 (81.8)	51 (77.3)	
Modified intention-to-treat	3 (13.6)	8 (12.1)	
Per-protocol	1 (4.6)	5 (7.6)	
Industry sponsor	19 (82.6)	51 (78.5)	0.90
Sponsor involved in trial design	8 (34.8)	17 (26.2)	0.60
Sponsor involved in statistical analysis	5 (21.7)	14 (21.5)	>0.99
Study results			
Noninferiority claiming	15 (68.2)	57 (86.4)	0.111
Percentage difference between anticipated and observed event rates	6.7 (−57.4 to 25.7)	18.4 (−4.8 to 37.5)	0.105
Study dissemination			
Conference			0.150
American College of Cardiology	9 (40.9)	14 (21.2)	
American Heart Association	2 (9.1)	6 (9.1)	
European Society of Cardiology	6 (27.3)	14 (21.2)	
Transcatheter Cardiovascular Therapeutics	5 (22.7)	32 (48.5)	
Journal			0.058
New England Journal of Medicine	13 (59.1)	19 (28.8)	
The Lancet	2 (9.1)	13 (19.7)	
Journal of the American Medical Association	2 (9.1)	3 (4.5)	
European Heart Journal	0 (0.0)	7 (10.6)	
Circulation	1 (4.5)	8 (12.1)	
JACC	0 (0.0)	5 (7.6)	
JACC: Cardiovascular Interventions	0 (0.0)	5 (7.6)	
Others	4 (18.2)	6 (9.1)	
Simultaneous publication	12 (52.2)	38 (58.5)	>0.99
Time from presentation to publication (days)^c^	134.5 (64.5-257.8)	262.0 (141.3-366.8)	0.094

Values are n (%) or median (IQR).

a The section 'Others' refers to trials not specifically focused on drug or device interventions. These include trials of therapeutic strategies, procedural approaches, and diagnostic or screening methodologies.

b Corresponding relative margin for trials using absolute metrics.

c Excluding simultaneously published trials.

**Central Illustration:**
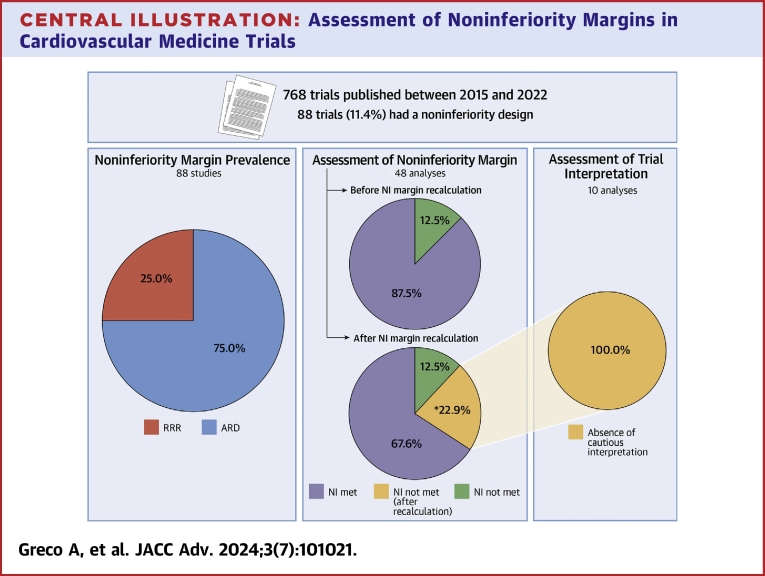
Assessment of Noninferiority Margins in Cardiovascular Medicine Trials Distribution of noninferiority randomized controlled trials with relative or absolute margin over the eligible population of studies. Number of included analyses who have met noninferiority before (eg, according to authors) and after the recalculation of noninferiority margin; Number prevalence of analyses not including cautionary notes of interpretation among studies with divergent results after the noninferiority margin recalculation. ∗Indicates 1 single trial that did not claim noninferiority in the original analysis, but met noninferiority criteria after recalculation, therefore being excluded from the assessment of study conclusions. ARD = absolute risk difference; NI = noninferiority; RRR = relative risk ratio.

### Assessment of noninferiority margin

After applying exclusion criteria, 48 analyses from 45 trials (ie, 3 trials reported 2 eligible analyses of co-primary endpoints) were eligible for recalculation of their noninferiority margins (Central Illustration). There were no significant differences in baseline characteristics between analyses eligible and not eligible to recalculation (Supplemental Table 3). In the eligible analyses, noninferiority was declared in 42 out of 48 (87.5%).

**Figure 2:**
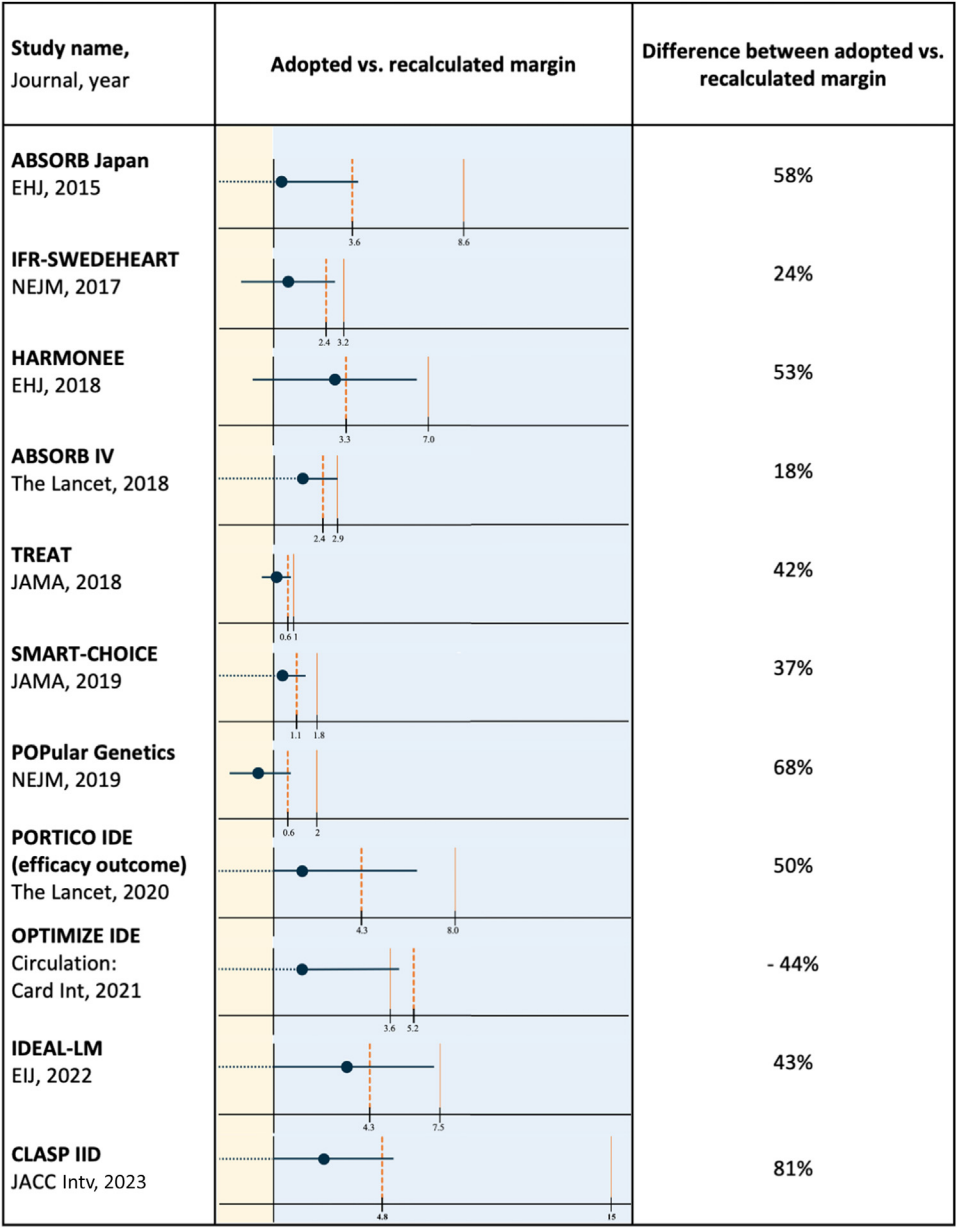
**Trials Showing Divergent Results After the Recalculation of Noninferiority Margin** Dashed orange lines refer to noninferiority margins based on the anticipated event rate in the control group; solid orange lines refer to recalculated noninferiority margins based on the observed event rate in the control group. Blue circles and lines represent absolute risk difference and CIs; light yellow and light blue stands for treatment better and control better areas, respectively. Circ Int = Circulation: Cardiovascular Interventions; EHJ = European Heart Journal; EIJ = EuroIntervention Journal; JACC Intv = JACC: Cardiovascular Interventions; JAMA = Journal of the American Medical Association; NEJM = New England Journal of Medicine; NI = noninferiority.

After the recalculation of the noninferiority margin, 31 analyses (64.6%) concluded for noninferiority consistently with the authors’ reporting, and 6 (12.5%) did not meet the noninferiority hypothesis consistently with the authors’ report. Divergent results were observed in 11 analyses (22.9%) (Table 2, Figure 2, Supplemental Table 4). Of these, 10 analyses originally claimed noninferiority but did not meet noninferiority after recalculation; conversely, 1 analysis initially rejected the noninferiority hypothesis, but noninferiority was re-established after recalculation, due to higher-than-anticipated event rates.^32^

**Table 2:** Characteristics of Trials Showing Divergent Results After the Recalculation of Noninferiority Margin

	Sample Size	Recalculated Sample Size	Study Design	Anticipated Event Rate (Control)	Primary Endpoint (Control)	Primary Endpoint (Treatment)	Primary Endpoint (RD)	ARD NI Margin	Corresponding RRR Margin	Recalculated ARD NI Margin	Difference Between Anticipated and Calculated ARD NI Margin
ABSORB Japan	400	2,166	Randomized, multicenter, single blinded	9.00%	5/133 (3.80%)	11/265 (4.20%)	0.4% (UCL: 3.95%)	8.60%	1.95	3.60%	58%
EHJ, 2015											
IFR-SWEDEHEART	2,037	3,388	Randomized, multicenter, open label	8.00%	61/1,007 (6.10%)	68/1,012 (6.70%)	0.7% (UCL: 2.80%)	3.20%	1.40	2.40%	24%
NEJM, 2017											
HARMONEE	572	1,916	Randomized, multicenter, open label	9.00%	12/285 (4.20%)	20/287 (7.00%)	2.8% (UCL: 6.50%)	7.00%	1.77	3.30%	53%
EHJ, 2018											
ABSORB IV	2,604	3,666	Randomized, multicenter, double blinded	4.90%	48/1,303 (3.70%)	64/1,288 (5.00%)	1.3% (UCL: 2.89%)	2.90%	1.54	2.40%	18%
The Lancet, 2018											
TREAT	3,799	12,582	Randomized, multicenter, single blinded	1.20%	13/1,886 (0.69%)	14/1,913 (0.73%)	0.04% (UCL: 0.58%)	1.00%	1.83	0.60%	42%
JAMA, 2018											
SMART-CHOICE	2,993	9,964	Randomized, multicenter, open label	4.00%	36/1,498 (2.50%)	42/1,495 (2.90%)	0.40% (UCL: 1.30%)	1.80%	1.45	1.10%	37%
JAMA, 2019											
POPular Genetics NEJM, 2019	2,488	104,868	Randomized, multicenter, single blinded	18.80%	73/1,246 (5.90%)	63/1,242 (5.10%)	−0.7% (UCL: 0.70%)	2.00%	1.10	0.60%	68%
PORTICO IDE (efficacy outcome)	750	2,508	Randomized, multicenter, open label	25.00%	48/369 (13.40%)	55/381 (14.80%)	1.5% (UCL: 6.50%)	8.00%	1.32	4.30%	50%
The Lancet, 2020											
OPTIMIZE IDE^a^	1,639	706	Randomized, multicenter, single blinded	6.50%	74/780 (9.50%)	82/796 (10.30%)	0.81% (UCL: 3.78%)	3.60%	1.55	5.20%	−44%
Circulation:											
Card Int, 2021											
IDEAL-LM	818	2,490	Randomized, multicenter, open label	20.00%	45/410 (11.40%)	59/408 (14.60%)	3.28% (UCL: 7.18%)	7.50%	1.37	4.30%	43%
EIJ, 2022											
CLASP IID	180	1,504	Randomized, multicenter, open label	25.00%	3/63 (4.80%)	4/117 (3.40%)	1.3% (UCL: 5.10%)	15%	1.60	2.88%	81%
JACC Intv											

ARD = absolute risk difference; Circ Int = Circulation: Cardiovascular Interventions; EHJ = European Heart Journal; EIJ = EuroIntervention Journal; JACC Intv = JACC: Cardiovascular Interventions; JAMA = Journal of the American Medical Association; NI = noninferiority; NEJM = New England Journal of Medicine; RD = risk difference; UCL = upper confidence limit.

a The OPTIMIZE IDE trial represents a notable exception in our study, showcasing a unique case where the interpretation was reversed after margin recalculation, contrasting with the direction of divergences observed in other trials with primary outcome discrepancies. In this study 1,639 patients were randomized to either a low-profile, fixed-wire drug-eluting stent or a conventional drug-eluting stent. The investigators anticipated a target lesion failure rate (primary endpoint) at 1 year of 6.5% in the control arm and defined an ARD margin of noninferiority of 3.58% (corresponding to a RRR margin of 1.55). The difference in the primary endpoint rate between the 2 treatments was 0.08%, with an upper confidence limit of 3.8%: noninferiority was not met and the trial was reported as failing the noninferiority hypothesis. However, several considerations may challenge the interpretation of this trial. Indeed, the higher-than-anticipated observed event rate in the control arm (9.5% instead of 6.5%) pushed the noninferiority margin up to 5.2%, theoretically enabling a claim of noninferiority.

### Assessment of trial interpretation

All the 10 analyses that originally claimed noninferiority but did not meet noninferiority after recalculation did not include cautionary notes to account for the chance of a differing interpretation (Central Illustration). Compared to others, these trials displayed a larger median difference between anticipated and recalculated noninferiority margins (44.7% [IQR: 38.6%-56.7%] vs 15.3% [IQR: −1.5% to 28.9%]; *P* < 0.001).

## Discussion

The main findings of this study can be summarized as follows: 1) 3 noninferiority trials out of 4 used an ARD margin; 2) in the analyses using an ARD, almost 1 out of 4 had different results after recalculating the noninferiority margin; and 3) notes of cautionary interpretation were lacking in the conclusions of all the analyses that originally claimed noninferiority but did not meet noninferiority after recalculation.

Noninferiority trials have been increasingly performed over the last decades, and a rigorous methodology is of utmost importance to avoid the inappropriate adoption of a treatment that may threat the outcomes of patients.^31^ For instance, “biocreep” is a detrimental process that can lead to the acceptance of an inadequate treatment as a result of a stepwise sequence of noninferiority proofs entailing a gradual loss of treatment effect.^33^ To standardize and improve the architecture of noninferiority trials, several pillars have been identified by experts and regulatory authorities: 1) determination of the noninferiority margin based on the results of previous placebo-controlled trials of the active control; 2) choice of a noninferiority margin scale (ie, absolute or relative); 3) selection of appropriate endpoints (eg, clinical relevance, availability of historical data); 4) assay sensitivity over placebo (ie, superiority of the active control to placebo); 5) trial conduct (eg, adequateness of treatment administration, endpoint adjudication); and 6) selection of data analysis (eg, intention-to-treat, per-protocol, as treated).^18^

Small observational studies and their meta-analyses highlighted critical issues regarding the scale (ie, absolute or relative) or calculation of the noninferiority margin.^24^, ^25^, ^26^, ^27^, ^28^, ^29^, ^30^ Regulatory authorities recommend to prioritize the use of a relative instead of an absolute margin, which is particularly desirable when there is a risk for a lower-than-anticipated event rate or the event rate is unpredictable.^20^^,^^21^ Relative metrics are advantageous at the population level (eg, to assess the effects of a treatment compared to a previous one) and are dimensionless (ie, do not numerically increase over observation periods, therefore remaining comparable regardless of the timing of the assessment).^22^^,^^23^ However, since they can be clinically less meaningful, particularly in the assessment of infrequent events, absolute metrics are preferred to express and communicate risks at the individual-patient level despite being susceptible to influence from individual factors (eg, health status, risk factors, comorbidities).^22^^,^^23^

Choosing an ARD conveys some risks of wrongly rejecting the null hypothesis (ie, inferiority of the experimental arm) if the trial terminates with a lower-than-anticipated event rate.^34^ This is due to a calibration inaccuracy that occurs when the calculation relies mathematically on a difference rather than on a ratio (ie, the difference is influenced by the magnitude of event rates, while the ratio is dimensionless and therefore avoids any distortion).

Although some investigations on the reporting of noninferiority trials have already been performed in other areas of medicine, they are limited in the cardiovascular field, and the accuracy of their interpretation has not been systematically investigated. In particular, the focus of our study was on interpretation and reporting of noninferiority trials in cardiovascular medicine. Recently, the reporting of noninferiority trials has been analyzed in the field of coronary interventions, where about 1 out of 3 studies claiming noninferiority based on an ARD displayed different results after recalculation.^30^ This is higher than in our study, which focused on a broader area of interest, including trials of drugs, devices and strategies in cardiovascular medicine. The use of different inclusion criteria is likely responsible for the lower proportion of divergent results after recalculation in our study.

Among trials with divergent results after margin recalculation in our series, one showed an inverse discordance due to a higher-than-anticipated event rate.^32^ This case represents an example of why the recalculation of the ARD noninferiority margin can be important also in case of higher-than-anticipated event rates. Indeed, underestimation of event rates may lead a treatment that is actually noninferior to the control to be erroneously declared inferior, not published, and eventually excluded from further clinical development.

The absence of statements of cautionary interpretation of the study results, unduly highlighting the experimental treatment as beneficial despite fragile statistically significance in the primary outcome, can present with a variety of forms, including an excess focus on statistically significant results with selective or incomplete reporting, empowerment of secondary or subgroup analyses, interpretation of statistically nonsignificant results as a proof of equivalence, or emphasis on beneficial effects despite a lack of statistical significance. Importantly, the absence of cautionary interpretation should not be necessarily considered as a fraudulent conduct since it can also arise from unconscious bias toward or against a treatment, or insufficient methodological knowledge. Consequently, our study underscores that the noninferiority setting involves multiple considerations beyond the noninferiority margin, and it emphasizes that a rigorous interpretation of these studies requires a comprehensive analysis of all evidence, especially in cases of significant deviations between expected and observed event rates. A large investigation on the interpretation of study results has been conducted among negative or neutral oncology noninferiority trials, where it affected 3 studies out of four.^24^ In our study, a lack of phrasing suggesting the need for cautionary interpretation was identified in all the analyses showing divergent results after the margin recalculation. Interestingly, the high median percentage difference between the prespecified and the recalculated margins in analyses with divergent results seemed to be rooted in the variance between anticipated and observed event rates found in these studies. This highlights a growing challenge in designing randomized trials, especially in rapidly advancing fields where interventions improve quickly, thus affecting performance and reducing adverse events, which are key trial endpoints. In fact inaccuracies in study design predispose to the change of study results when the noninferiority margin is recalculated, therefore substantially increasing the likelihood of claiming noninferiority based on inappropriate assumptions.^20^^,^^21^

To the best of our knowledge, this is the first study to provide a comprehensive appraisal of critical issues related to noninferiority trials in cardiovascular medicine with a focus on the assessment of the noninferiority margin. Our findings imply that Authors, Reviewers and Editors should be aware of the risks of inappropriate reporting and interpretation of study results when dealing with designing, reporting, interpreting, and commenting on noninferiority trials, particularly if absolute metrics are used or in case of large discrepancy between observed and anticipated event rates in the control arm.

### Study Limitations

We acknowledge some limitations of this analysis. First, the lower-than-anticipated event rates may be influenced by confounders, including a higher-than-anticipated efficacy of the active control; however, we focused our analysis on the presence and impact of a lower-than-anticipated event rate, regardless of its causal mechanism. Second, our recalculated noninferiority margin is modified in a data-driven way, therefore our method might be exposed to a certain grade of type I error risk inflation, and it cannot be recommended universally. To this purpose, Quartagno et al^35^^,^^36^ proposed the concept of power-stabilizing noninferiority boundaries to handle unexpected event risk in the control group. Third, we did not assess the impact of study misinterpretations on regulatory approvals or guideline formulations, a complex area beyond the scope of this observational cohort study. Finally, while observer bias during full-text assessments of peer-reviewed manuscripts cannot be entirely excluded for the secondary endpoint investigation, it was mitigated by using a standardized and reproducible mathematical calculation for the primary endpoint.

## Conclusions

Randomized clinical trials are one of the most acknowledged and trusted sources of knowledge in current evidence-based medicine. A number of issues can impair the proper reporting of a trial, including endpoint selection, assay sensitivity over placebo, adequateness of trial conduct, and selection of a proper data analysis plan. An additional peculiar challenge when dealing with noninferiority trials is represented by the determination of the type and magnitude of the noninferiority margin. Recalculating noninferiority margins based on actual event rates led to different outcomes in 1 out of 4 cardiovascular trials, and most differing trials lacked cautionary interpretation. To minimize these risks, recommendations from regulatory authorities should be highly regarded when conceiving, designing, conducting, reporting, and interpreting a noninferiority trial.

Perspectives **COMPETENCY IN MEDICAL KNOWLEDGE** Noninferiority trials are becoming more common in cardiovascular medicine, but their reporting and interpretation can be difficult, particularly when using an absolute risk difference as the noninferiority margin. In cardiovascular medicine, absolute metrics are frequently preferred; however, using an absolute margin may carry some risk of regression towards noninferiority if the trial terminates with a lower-than-anticipated event rate. The adoption of both absolute and relative noninferiority margins might be the safest solution to address this issue.

**TRANSLATIONAL OUTLOOK:** Among noninferiority trials in cardiovascular medicine, the majority adopted an ARD margin and almost 1 out of 4 presented divergent results after recalculating the noninferiority margin. Most trials did not include notes of cautionary interpretation in the conclusions section. Our findings imply that Authors, Reviewers and Editors should be aware of the risks related to the choice of the noninferiority margin when dealing with designing, reporting, interpreting, and commenting on noninferiority trials, particularly if absolute metrics are used and in case of large discrepancy between observed and anticipated event rates in the control arm.

## Funding support and author disclosures

Dr Capodanno has received honoraria from Novo Nordisk, Sanofi and Terumo, and Institutional fees from Medtronic. All other authors have reported that they have no relationships relevant to the contents of this paper to disclose.

## References

[1] Collins R., MacMahon S. (2001). Reliable assessment of the effects of treatment on mortality and major morbidity, I: clinical trials. Lancet.

[2] Zelen M. (1979). A new design for randomized clinical trials. N Engl J Med.

[3] Concato J., Shah N., Horwitz R.I. (2000). Randomized, controlled trials, observational studies, and the Hierarchy of research designs. N Engl J Med.

[4] Ellenberg S.S. (2000). Placebo-controlled trials and active-control trials in the evaluation of new treatments. Part 2: Practical issues and specific cases. Ann Intern Med.

[5] Kates A.M., Morris P., Poppas A., Kuvin J.T. (2018). Impact of live, Scientific annual meetings in Today’s cardiovascular World. J Am Coll Cardiol.

[6] Mylotte D., Byrne R. (2017). EuroPCR 2017, late-breaking clinical trials and EuroIntervention. EuroIntervention.

[7] Seto A.H., Safirstein J., Anwaruddin S., Dehghani P., Shah B., Tremmel J.A. (2016). Late breaking trials of 2015 in coronary artery disease: commentary covering ACC, EuroPCR, SCAI, TCT, ESC, and AHA. Catheter Cardiovasc Interv.

[8] Seto A.H., Dehghani P., Shah B., Anwaruddin S., Safirstein J., Tremmel J.A. (2017). Late breaking trials of 2016 in coronary artery disease: Commentary covering SCAI, ACC, TCT, EuroPCR, ESC, and AHA. Catheter Cardiovasc Interv.

[9] Medranda G.A., Case B.C., Wermers J.P. (2021). Review of interventional late breaking trials from AHA Scientific sessions 2020 Virtual meeting. Cardiovasc Revasc Med.

[10] Spagnolo M., Greco A., Laudani C. (2023). Association of trial characteristics with simultaneous publication and its impact on citations and mentions: a cross-sectional study. Rev Esp Cardiol (Engl Ed).

[11] Boutron I. (2020). Spin in Scientific publications: a frequent detrimental research practice. Ann Emerg Med.

[12] Arunachalam L., Hunter I.A., Killeen S. (2017). Reporting of randomized controlled trials with statistically nonsignificant primary outcomes published in high-impact surgical journals. Ann Surg.

[13] Reynolds-Vaughn V., Riddle J., Brown J., Schiesel M., Wayant C., Vassar M. (2020). Evaluation of spin in the abstracts of Emergency medicine randomized controlled trials. Ann Emerg Med.

[14] Rassy N., Rives-Lange C., Carette C. (2021). Spin occurs in bariatric surgery randomized controlled trials with a statistically nonsignificant primary outcome: a systematic review. J Clin Epidemiol.

[15] Wang D., Chen L., Wang L. (2021). Abstracts for reports of randomized trials of COVID-19 interventions had low quality and high spin. J Clin Epidemiol.

[16] Garrett M., Koochin T., Ottwell R. (2021). Evaluation of spin in the abstracts of systematic reviews and meta-analyses of treatments and interventions for smoking cessation. Tob Prev Cessat.

[17] Pereira G.C., Prates G., Medina M. (2021). High frequency of spin bias in controlled trials of cannabis derivatives and their synthetic analogues: a meta-epidemiologic study. J Clin Epidemiol.

[18] Mauri L., D'Agostino RBSr (2017). Challenges in the design and interpretation of noninferiority trials. N Engl J Med.

[19] Kaul S. (2021). Understanding the Merits and Drawbacks of noninferiority trials in cardiovascular medicine. Can J Cardiol.

[20] U.S. Department of Health and Human Services, Food and Drug Administration, Center for Drug Evaluation and Research (CDER), Center for Biologics Evaluation and Research (CBER) (2016).

[21] Committee for medicinal products for human use (CHMP) (2005).

[22] Pocock S.J. (2003). The pros and cons of noninferiority trials. Fundam Clin Pharmacol.

[23] Head S.J., Kaul S., Bogers A.J.J.C., Kappetein A.P. (2012). Non-inferiority study design: lessons to be learned from cardiovascular trials. Eur Heart J.

[24] Ito C., Hashimoto A., Uemura K., Oba K. (2021). Misleading reporting (spin) in noninferiority randomized clinical trials in oncology with statistically not significant results. JAMA Netw Open.

[25] He Y., Shu C., Li T. (2021). Non-inferiority in cancer clinical trials was associated with more lenient margins and higher hypothesized outcome event rates. J Clin Epidemiol.

[26] Aupiais C., Zohar S., Taverny G., Le Roux E., Boulkedid R., Alberti C. (2018). Exploring how non-inferiority and equivalence are assessed in paediatrics: a systematic review. Arch Dis Child.

[27] Rief W., Hofmann S.G. (2018). Some problems with non-inferiority tests in psychotherapy research: psychodynamic therapies as an example. Psychol Med.

[28] Golish S.R. (2017). Pivotal trials of orthopedic surgical devices in the United States: predominance of two-arm non-inferiority designs. Trials.

[29] Charlesworth M., Choi S.W. (2018). Non-inferiority studies: is ‘better’ the enemy of ‘good enough’?. Anaesthesia.

[30] Simonato M., Ben-Yehuda O., Vincent F., Zhang Z., Redfors B. (2022). Consequences of inaccurate assumptions in coronary stent noninferiority trials. JAMA Cardiol.

[31] Danielsen A.K., Okholm C., Pommergaard H.C., Burcharth J., Rosenberg J. (2014). Number of published randomized controlled multi center trials testing pharmacological interventions or devices is increasing in both medical and surgical specialties. PLoS One.

[32] Kereiakes D.J., Feldman R.L., Ijsselmuiden A.J.J. (2021). Safety and Effectiveness of the SVELTE fixed-wire and Rapid Exchange Bioresorbable-Polymer Sirolimus-eluting coronary stent Systems for the treatment of Atherosclerotic lesions: results of the OPTIMIZE randomized study. Circ Cardiovasc Interv.

[33] Everson-Stewart S., Emerson S.S. (2010). Bio-creep in non-inferiority clinical trials. Stat Med.

[34] Bai A.D., Komorowski A.S., Lo C.K.L. (2021). Intention-to-treat analysis may be more conservative than per protocol analysis in antibiotic non-inferiority trials: a systematic review. BMC Med Res Methodol.

[35] Li Z., Quartagno M., Bohringer S., van Geloven N. (2022). Choosing and changing the analysis scale in non-inferiority trials with a binary outcome. Clin Trials.

[36] Quartagno M., Walker A.S., Babiker A.G. (2020). Handling an uncertain control group event risk in non-inferiority trials: non-inferiority frontiers and the power-stabilising transformation. Trials.

