# Supplementary material for: Assessment of Noninferiority Margins in Cardiovascular Medicine Trials
Source: JACC Adv. 2024 Jun 5;3(7):101021. doi: 10.1016/j.jacadv.2024.101021 (PMC11312784; doi:10.1016/j.jacadv.2024.101021)
Supplement: Supplemental Data [file mmc1.docx]

**SUPPLEMENTAL APPENDIX**

**Supplemental Methods 1 – Search strategy**

1. Scientific programs of selected conferences (i.e., American College of Cardiology, American Heart Association, European Society of Cardiology and Transcatheter Cardiovascular Therapeutics) from 2015 to 2022 were searched for in official websites of each corresponding organization and then double checked with information reported in websites of divulgation in cardiovascular medicine.
2. Late breaking science sessions were scrutinized for each conference in search of eligible randomized trials.
3. For each study presented, the corresponding published article was searched in PubMed using the title of presentation and name of the presenter as research terms.
4. The temporal relation between presentation and publication was checked and, in doubtful cases (e.g., studies published before the presentation, study published after at least 100 days from presentation, subgroup analyses), the study design and protocol were consulted in the official trial registration website (e.g., clinicaltrials.gov) using the identification code.
5. Eligible studies were independently examined by two investigators at the full-text level to apply the inclusion and exclusion criteria; eventual disagreements were solved by consensus.
6. A final list of eligible studies was drafted and approved by consensus.

**Supplemental Methods 2 – Stepwise numerical example of noninferiority margin assessment**

The ABSORB Japan trial compared the Absorb (Abbott Vascular) bioabsorbable vascular stent to the Xience (Abbott Vascular) cobalt-chromium everolimus-eluting stent in terms of target lesion failure at 12 months (primary endpoint).

The investigators anticipated an event rate in the control group of 9.0% and prespecified an absolute noninferiority margin of 8.6%. At 12 months, the primary endpoint occurred in 3.8% and 4.2% of patients in the control and investigational arms, respectively, resulting into an upper confidence limit of the absolute risk difference of 3.95%. The investigators claimed noninferiority of the Absorb bioabsorbable vascular stent to the cobalt-chromium everolimus-eluting stent because the upper confidence limit of the absolute risk difference (3.95%) did not cross the noninferiority margin (8.6%).

We assessed the noninferiority margin in this trial as follows:

1. Determine the acceptable event rate as the sum of anticipated event rate in the control group (9.0%) and absolute risk difference non-inferiority margin (8.6%):

*9.0% + 8.6% = 17.6%*

1. Estimate the corresponding relative margin as the ratio of acceptable event rate (17.6%) and anticipated event rate (9.0%):

*17.6% / 9.0% = 1.95*

1. Determine the acceptable event rate according to the relative margin as the product of corresponding relative margin (1.95) and observed event rate in the control group (3.8%):

*1.95* x *3.8% = 7.4%*

1. Calculate the adjusted absolute non-inferiority margin as the difference between acceptable event rate according to relative margin (7.4%) and observed event rate in the control group (3.8%):

*7.4% – 3.8% = 3.6%*

After such re-calculation of the noninferiority margin, noninferiority was not met because the upper confidence limit of the absolute risk difference (3.95%) crossed the recalculated noninferiority margin (3.6%).

**Supplemental Table 1 –** STROBE checklist of items that should be included in reports of observational studies

|  | Item No | Recommendation | Page(s) |
| --- | --- | --- | --- |
| **Title and abstract** | 1 | (*a*) Indicate the study’s design with a commonly used term in the title or the abstract | 1 |
|  |  | (*b*) Provide in the abstract an informative and balanced summary of what was done and what was found | 3 |
| Introduction | | |  |
| Background/rationale | 2 | Explain the scientific background and rationale for the investigation being reported | 4-5 |
| Objectives | 3 | State specific objectives, including any prespecified hypotheses | 6 |
| Methods | | |  |
| Study design | 4 | Present key elements of study design early in the paper | 6 |
| Setting | 5 | Describe the setting, locations, and relevant dates, including periods of recruitment, exposure, follow-up, and data collection | 7 |
| Participants | 6 | (*a*) *Cohort study*—Give the eligibility criteria, and the sources and methods of selection of participants. Describe methods of follow-up  *Case-control study*—Give the eligibility criteria, and the sources and methods of case ascertainment and control selection. Give the rationale for the choice of cases and controls  *Cross-sectional study*—Give the eligibility criteria, and the sources and methods of selection of participants | 6 |
|  |  | (*b*) *Cohort study*—For matched studies, give matching criteria and number of exposed and unexposed  *Case-control study*—For matched studies, give matching criteria and the number of controls per case | 6-7 |
| Variables | 7 | Clearly define all outcomes, exposures, predictors, potential confounders, and effect modifiers. Give diagnostic criteria, if applicable | 7-8 |
| Data sources/ measurement | 8* | For each variable of interest, give sources of data and details of methods of assessment (measurement). Describe comparability of assessment methods if there is more than one group | *6* |
| Bias | 9 | Describe any efforts to address potential sources of bias | 15-16 |
| Study size | 10 | Explain how the study size was arrived at | 6 |
| Quantitative variables | 11 | Explain how quantitative variables were handled in the analyses. If applicable, describe which groupings were chosen and why | 8 |
| Statistical methods | 12 | (*a*) Describe all statistical methods, including those used to control for confounding | 8-9 |
|  |  | (*b*) Describe any methods used to examine subgroups and interactions | 8-9 |
|  |  | (*c*) Explain how missing data were addressed | 7 |
|  |  | (*d*) *Cohort study*—If applicable, explain how loss to follow-up was addressed  *Case-control study*—If applicable, explain how matching of cases and controls was addressed  *Cross-sectional study*—If applicable, describe analytical methods taking account of sampling strategy |  |
|  |  | (*e*) Describe any sensitivity analyses |  |
| Results |  |  |  |
| Participants | 13* | (a) Report numbers of individuals at each stage of study—eg numbers potentially eligible, examined for eligibility, confirmed eligible, included in the study, completing follow-up, and analysed | 9 |
|  |  | (b) Give reasons for non-participation at each stage | 9 |
|  |  | (c) Consider use of a flow diagram | 9 |
| Descriptive data | 14* | (a) Give characteristics of study participants (eg demographic, clinical, social) and information on exposures and potential confounders | 9 |
|  |  | (b) Indicate number of participants with missing data for each variable of interest | 9 |
|  |  | (c) *Cohort study*—Summarise follow-up time (eg, average and total amount) |  |
| Outcome data | 15* | *Cohort study*—Report numbers of outcome events or summary measures over time |  |
|  |  | *Case-control study—*Report numbers in each exposure category, or summary measures of exposure |  |
|  |  | *Cross-sectional study—*Report numbers of outcome events or summary measures | 10-11 |
| Main results | 16 | (*a*) Give unadjusted estimates and, if applicable, confounder-adjusted estimates and their precision (eg, 95% confidence interval). Make clear which confounders were adjusted for and why they were included | 10-11 |
|  |  | (*b*) Report category boundaries when continuous variables were categorized | 10-11 |
|  |  | (*c*) If relevant, consider translating estimates of relative risk into absolute risk for a meaningful time period |  |
| Other analyses | 17 | Report other analyses done—eg analyses of subgroups and interactions, and sensitivity analyses |  |
| Discussion |  |  |  |
| Key results | 18 | Summarise key results with reference to study objectives | 11 |
| Limitations | 19 | Discuss limitations of the study, taking into account sources of potential bias or imprecision. Discuss both direction and magnitude of any potential bias | 15-16 |
| Interpretation | 20 | Give a cautious overall interpretation of results considering objectives, limitations, multiplicity of analyses, results from similar studies, and other relevant evidence | 12-13 |
| Generalisability | 21 | Discuss the generalisability (external validity) of the study results | 14-15 |
| Other information |  |  |  |
| Funding | 22 | Give the source of funding and the role of the funders for the present study and, if applicable, for the original study on which the present article is based | 1 |

**Supplemental Table 2 –** Baseline characteristics of the identified randomized noninferiority trials.

|  | **Noninferiority trials (n=88)** |
| --- | --- |
| **Study design** | |
| Topic – n (%) | |
| Devices | 52 (59.1) |
| Drugs | 27 (30.7) |
| Others | 9 (10.2) |
| Multicentre – n (%) | 86 (97.7) |
| Trial design – n (%) | |
| Open label | 62 (70.5) |
| Single blind | 19 (21.6) |
| Double blind | 7 (8.0) |
| Sample size – median (IQR) | 1,504 (761.0-2,471.5) |
| Follow-up (weeks) – median (IQR) | 52.1 (34.6-56.5) |
| Type of analysis – n (%) | |
| As treated | 2 (2.3) |
| Intention-to-treat | 69 (78.4) |
| Modified intention-to-treat | 11 (12.5) |
| Per-protocol | 6 (6.8) |
| Industry sponsor – n (%) | 70 (79.5) |
| Sponsor involved in trial design – n (%) | 25 (28.4) |
| Sponsor involved in statistical analysis – n (%) | 19 (21.6) |
| **Study results** | |
| Noninferiority claiming – n (%) | 72 (81.8) |
| Percentage difference between anticipated and  observed event rates – median % (IQR) | 17.4 (– 9.7-35.9) |
| **Study dissemination** | |
| Conference – n (%) | |
| American College of Cardiology | 23 (26.1) |
| American Heart Association | 8 (9.1) |
| European Society of Cardiology | 20 (22.7) |
| Transcatheter Cardiovascular Therapeutics | 37 (42.1) |
| Journal - n (%) | |
| New England Journal of Medicine | 32 (36.4) |
| The Lancet | 15 (17.0) |
| Journal of the American Medical Association | 5 (5.7) |
| European Heart Journal | 7 (8.0) |
| Circulation | 9 (10.2) |
| JACC | 5 (5.7) |
| JACC: Cardiovascular Interventions | 5 (5.7) |
| Others | 10 (11.4) |
| Simultaneous publication – n (%) | 50 (56.8) |
| Time from presentation to publication (days)* – median (IQR) | 235.0 (120.2-333.5) |

* Excluding simultaneously published trials. Abbreviations: IQR, Interquartile Range; n, number; JACC, Journal of American College of Cardiology.

**Supplemental Table 3 –** Characteristics of the randomised noninferiority trials eligible and those not eligible to the analysis of noninferiority margins.

|  | **Eligible**  **(n=45)** | **Not eligible (n=43)** | **P value** |
| --- | --- | --- | --- |
| **Study design** |  | | |
| Topic – n (%) |  |  | 0.28 |
| Devices | 30 (66.7) | 22 (51.1) |  |
| Drugs | 12 (26.7) | 15 (34.9) |  |
| Others | 3 (6.6) | 6 (14.0) |  |
| Multicentre – n (%) | 45 (100.0) | 41 (95.3) | 0.45 |
| Trial design – n (%) |  |  | 0.143 |
| Open label | 30 (66.7) | 32 (74.4) |  |
| Single blind | 13 (28.9) | 6 (14.0) |  |
| Double blind | 2 (4.4) | 5 (11.6) |  |
| Sample size – median (IQR) | 1,639  (880-2,488) | 1,426  (496-2,338.5) | 0.28 |
| Follow-up (weeks) – median (IQR) | 52.1 (52.0-52.1) | 52.1 (29.2-80.9) | 0.51 |
| Type of analysis – n (%) |  |  | 0.42 |
| As treated | 1 (2.2) | 1 (2.3) |  |
| Intention-to-treat | 33 (73.4) | 36 (83.7) |  |
| Modified intention-to-treat | 6 (13.3) | 5 (11.7) |  |
| Per-protocol | 5 (11.1) | 1 (2.3) |  |
| Industry sponsor – n (%) | 37 (82.2) | 33 (76.7) | 0.71 |
| Sponsor involved in trial design – n (%) | 13 (28.9) | 12 (27.9) | >0.99 |
| Sponsor involved in statistical analysis – n (%) | 11 (24.4) | 8 (18.6) | 0.68 |
| **Study results** |  |  |  |
| Noninferiority claiming – n (%) | 39 (86.7) | 33 (76.7) | 0.35 |
| Percentage difference between anticipated and  observed event rates – median % (IQR) | 21.7 (2.0-36.0) | 6.9 (– 33.7-30.3) | 0.089 |
| **Study dissemination** |  |  |  |
| Conference – n (%) |  |  | 0.58 |
| American College of Cardiology | 11 (24.4) | 12 (27.9) |  |
| American Heart Association | 5 (11.1) | 3 (7.0) |  |
| European Society of Cardiology | 8 (17.8) | 12 (27.9) |  |
| Transcatheter Cardiovascular Therapeutics | 21 (46.7) | 16 (37.2) |  |
| Journal - n (%) |  |  | 0.46 |
| New England Journal of Medicine | 17 (37.8) | 15 (34.9) |  |
| The Lancet | 10 (22.2) | 5 (11.6) |  |
| Journal of the American Medical Association | 3 (6.7) | 2 (4.6) |  |
| European Heart Journal | 3 (6.7) | 4 (9.3) |  |
| Circulation | 6 (13.3) | 3 (7.0) |  |
| JACC | 1 (2.2) | 4 (9.3) |  |
| JACC: Cardiovascular Interventions | 2 (4.4) | 3 (7.0) |  |
| Others | 3 (6.7) | 7 (16.3) |  |
| Simultaneous publication – n (%) | 28 (62.2) | 22 (51.2) | 0.41 |
| Time from presentation to publication (days)* – median (IQR) | 272.0  (159.0-341.0) | 181.0  (108.0-318.5) | 0.33 |

Abbreviations: IQR, Interquartile Range; n, number; JACC, Journal of American College of Cardiology.

**Supplemental Table 4 –** Detailed noninferiority architecture of trials showing divergent results after the recalculation of noninferiority margin.

| **TRIAL**  Journal, Year | **Endpoint**  (timeframe) | **Historical control event rate (justification or source)** | **Historical control baseline characteristics** | **Estimated active control** | **Control group baseline characteristics** | **Primary endpoint: Control** | **Primary endpoint:**  **Treatment group** | **Primary endpoint CI UCL** | **ARD NI margin** |
| --- | --- | --- | --- | --- | --- | --- | --- | --- | --- |
| **ABSORB JAPAN** EHJ, 2015 | Target lesion failure  (12 months) | 8.6%  (half of the estimated TTE) | NA | 9.0% | Age (mean, SD): 67.3±9.6  Diabetes (%): 35.8  UA (%): 16.4 | 5/133  (3.80%) | 11/265  (4.20%) | 3.95% | 8.60% |
| **IFR-SWEDEHEART**  NEJM, 2017 | Composite of all-cause death, nonfatal MI, or unplanned revascularization  (12 months) | 8.0%  (SWEEDHEART registry) | NA | 8.0% | Age (mean, SD): 67.4±9.2  Diabetes (%): 20.9  Prior MI (%): 32.9  MVD (%): 9.9 | 61/1007  (6.10%) | 68/1012  (6.70%) | 2.80% | 3.20% |
| **HARMONEE**  Eur Heart J, 2018 | Target vessel failure  (12 months) | 9.8%  (Bern-Rotterdam) registry | Age (mean, SD): 65.3 ± 11.8  Diabetes (%): 18.1  NSTEMI (%): 32.9  MVD (%): 23.5 | 9.0% | Age (mean, SD): 66.5 (10.4)  Diabetes (%): 32.6  NSTEMI (%): 4.2  MVD (%): 10.9 | 12/285  (4.20%) | 20/287  (7.00%) | 6.50% | 7.00% |
| **ABSORB IV**  The Lancet, 2018 | Target lesion failure  (30 days) | 4.2 % (SPIRIT IV trial at 1 year) | Age (mean, SD): 63.3 ± 10.5  Diabetes (%): 32  UA (%): 27.7  RVD (%): 2.75±0.48 | 4.9% | Age (mean, SD): 62.2±10.3  Diabetes (%): 32  UA (%): 18  RVD (%): 2.89±0.38 | 48/1303  (3.70%) | 64/1288  (5.00%) | 2.89% | 2.90% |
| **TREAT**  JAMA, 2018 | Major bleeding  (30 days) | 1.2%  (ATLANTIC trial) | Age (mean, SD): 60.6 ± 12.4  Prior Stroke (%): 3.3* | 1.2% | Age (mean, IQR): 59 (51.6-65.2)  Prior Stroke (%): 4.3* | 13/1886  (0.69%) | 14/1913  (0.73%) | 0.58% | 1.00% |
| **SMART-CHOICE**  JAMA, 2019 | Major adverse cardiac and cerebrovascular events  (12 months) | 2.6%§  (EXCELLENT trial) | Age (mean, SD): 62.4±10.4  Diabetes (%): 32  CKD (%): 1.2  Previous MI (%): 3.7  STEMI (%): 38.6 | 4.0% | Age (mean, SD): 64.4±10.7  Diabetes (%): 36.8  CKD (%): 3.5  Previous MI (%): 4.3  STEMI (%): 3.6 | 36/1498  (2.50%) | 42/1495  (2.90%) | 1.30% | 1.80% |
| **POPular Genetics**  NEJM, 2019 | Net adverse clinical events (12 months) | 12.1%  (TRITON trial) | Age (mean): 61  Diabetes (%): 23  CKD (%): 12 | 18.8% | Age (mean, SD): 61.4±11.5  Diabetes (%): 11.1  CKD (%): 8.8 | 73/1246  (5.90%) | 63/1242  (5.10%) | 0.70% | 2.00% |
| **PORTICO (efficacy analysis)**  The Lancet, 2020 | All-cause mortality or disabling stroke  (12 months) | 24.2% (TVT registry) | Age (mean, SD): 80.9 ± 8.5  STS score (mean, SD): 7.4 ± 4.8  Previous stroke (%): 11.2  Porcelain aorta (n%): 4.4 | 25.0% | Age (mean, SD): 83.7±7  STS score (mean, SD): 6.6 ± 3.4  Previous stroke (%): 13.3  Porcelain aorta (n%): 2.7 | 48/369  (13.40%) | 55/381  (14.80%) | 6.50% | 8.00% |
| **OPTIMIZE**  Circulation: Card Interv 2021 | Target lesion failure  (12 months) | 6.9%^§^  (EVOLVE II trial) | Age (mean, SD): 63.5±10.4  Diabetes (%): 31.1  UA (%): 33.9  RVD (%):2.62±0.49  Mod. AHA/ACC B2/C (%): 76.8 | 6.5% | Age (mean, SD): 65.8±10.3  Diabetes (%): 30.7  UA (%): 25  RVD (%):2.77±0.50 | 74/780  (9.50%) | 82/796  (10.30%) | 3.78% | 3.60% |
| **IDEAL-LM**  Eurointervention, 2022 | Major adverse cardiac events  (2 years) | 21.3%^§^  (ISAAR-LM trial) | Age (mean, SD): 69.3±9.4  Diabetes (%): 28  ACS (%): 40 | 20.0% | Age (mean, SD): 66.0±10.5  Diabetes (%): 22.8  ACS (%): 40.2 | 45/410  (11.40%) | 59/408  (14.60%) | 7.18% | 7.50% |
| **CLASP IID**  JACC: Card Interv 2022 | Major adverse events (30 days) | NA | NA | 25.0% | Age (mean, SD): 81.2±6.2  CAD (%): 39.7  CKD (%): 42.9  Prior Stroke (%): 1.6  Prior MI (%): 11.1 | 3/63 (4.80%) | 4/117  (3.40%) | 1.3% | 15% |

Abbreviations: ACS, Acute coronary Syndrome; CAD, coronary artery disease; CKD, Chronic kidney Disease; LM: Left main; MACE, Major Adverse cardiovascular Events; MI, Myocardial infarction; MVD, Multivessel Disease; NA, Not Available; RCT, Randomized Controlled Trial; RVD, Reference Vessel Diameter; SD, standard deviation; STEMI, ST-segment elevation myocardial infarction; TTE, Total Treatment Effect; UA, Unstable Angina.
* additional baseline characteristics were not available for comparison
^§^ esteemed from the sum of single endpoints

**Supplemental Figure 1 –** Step-by-step assessment of noninferiority margin.

****For each analysis, the acceptable event rate (ER) was derived as the sum of the anticipated ER in the control group and the ARD NI margin. The corresponding RRR NI margin was then calculated as the ratio between the acceptable ER in the control and the anticipated event rate in the control arm. The RRR acceptable event rate was then calculated as the product of the corresponding RRR NI margin and the observed ER in the control arm of the study. Finally, an adjusted ARD NI margin was calculated as the difference between the RRR acceptable event rate and the observed ER in the control arm. Abbreviations: ARD, Absolute different difference noninferiority margin; ER, Event Rate; NI, Noninferiority; RRR, Relative Risk Ratio.
